# Performance Degradation Analysis of c-Si PV Modules Mounted on a Concrete Slab under Hot-Humid Conditions Using Electroluminescence Scanning Technique for Potential Utilization in Future Solar Roadways

**DOI:** 10.3390/ma12244047

**Published:** 2019-12-05

**Authors:** Firoz Khan, Jae Hyun Kim

**Affiliations:** 1 Center of Research Excellence in Renewable Energy (CORERE), King Fahd University of Petroleum & Minerals (KFUPM), Dhahran 31261, Saudi Arabia; 2 Energy Convergence Research Group, Daegu Gyeongbuk Institute of Science & Technology (DGIST), 333, Techno Jungang-Daero, Hyeonpung-Myeon, Dalseong-Gun, Daegu 42988, Korea

**Keywords:** renewable energy, PV module, solar roadways, PV degradation, electroluminescence scanning, PV cell parameters

## Abstract

The stability of the photovoltaic (PV) modules is critical when deployed in a non-ideal environment. Among the different factors, temperature and humidity are the two major factors affecting PV stability, making them significant causes of its degradation in terms of optoelectric and materials properties. Nowadays, with the increase in PV installation (here, we are only taking account of c-Si-based PV modules) to generate green electricity, effective space utilization is an important issue. Recently, people have been considering deploying PV modules on the road to utilize the space available on highways (roadways). This raises several new issues in the deployment of PV modules. However, issues related to temperature and humidity retain the same importance. Normally, these stability tests are performed in a damp-heat (DH) stress-testing chamber in an accelerated condition at 85 °C and 85% relative humidity (RH). In this work, c-Si PV modules were fixed over a concrete slab to prepare a PV interacted block, which can be used to build concrete-based roads. The performance of this PV on the concrete slab was tested in a DH stress-testing chamber in an accelerated condition at 85 °C and 85% RH for 4000 h. For the comparison, a PV module without concrete was also evaluated. The degradation of the PV modules was characterized using the electroluminescence scanning technique. After 2500 h of exposure to the DH conditions, the performance retention of the PV modules mounted on the concrete was 93.2%, which was nearly 5% higher than the module without the concrete slab.

## 1. Introduction

Crystalline silicon (c-Si) is dominating the PV market, because of its superior stability and performance, both of which affect the financial viability of solar PV installations. Normally, the PV modules pass through the stability certification under different simulated environmental conditions before commercialization. These procedures include damp*–*heat (DH), thermal cycling (TC), UV exposure, mechanical load, etc., as well as various other aging mechanisms [1]. However, for a highly specific application environment, this stability certification may not be sufficient to necessitate PV installation. Thus, it required specific study of the PV modules performances under that particular environment.

Again, since PV based power plants occupy considerable land space, several countries are considering utilizing the free space of roadways. This would mean the space could be used for transportation and power generation at the same time. However, degradation and reliability are key factors in the implementation of this plan. Among the various factors affecting the stability of the PV modules, temperature and damp are more present than ever.

Common c-Si PV modules available on the market are guaranteed to operate for ~25 years and still provide 80% of initial power after this period [2]. To meet this standard, PV modules have to survive 25 years of outdoor exposure under real environmental conditions. Since waiting for 25 years to obtain the data is unfeasible, several indoor characterization techniques have been recommended by the International Electrotechnical Commission (IEC) 61215 [2,3]. These procedures, including DH, TC, UV exposure, mechanical strength, etc., can be used to explore various aging mechanisms [1,2,3]. There are several reports available in the literature that deal with DH testing for estimating the reliability and lifetime of PV modules [1,4,5,6,7,8]. Regrettably, most of these tests simply determine whether the PV modules passed or failed the test. However, some of the papers deal with estimating the lifetime of the modules. For example, Hacke et al. [9] used DH testing, in situ dark current-voltage (*I–V*) measurement (biased at −600 V) and ex*–*situ illuminated *I–V* measurement at 100 mW/cm^2^ (25 °C) to estimate the degradation rate and predict lifetime. Zhu et al. [1] used various environmental conditions for the DH testing of three types of PV modules (with different types of back sheets). They [1] derived humidity dose from humidity, temperature, and time (1 dose ≈ 1430 h DH stress at 85 °C, 85% relative humidity, RH), and this data was then used to extract the power degradation, along with some of the solar cell parameters. Other research groups [1,2,10] have studied the degradation of some PV cell parameters (photogenerated current *I_ph_*, shunt resistance *R_sh_*, series resistance *R_s_*, diode ideality factor *n* and reverse saturation current *I_0_*) under DH stress. 

In this work, c-Si PV modules were mounted on the concrete slab, which can be used to study the effect of high temperature and high humidity on the contraction of roadways. As no similar study is available, the power degradation of PV mounted concrete slabs was monitored in a simulated environment (85 °C and 85% RH) in an accelerated condition for 4000 h, and data were collected at different time intervals. Degradation of the performance parameters such as short circuit current *I_sc_*, open-circuit voltage *V_oc_*, fill factor *FF* and efficiency *η* were compared with the unmounted PV module. Five analytical PV cell parameters were used to conduct a loss analysis. The degradation of the PV modules was also characterized using the electroluminescence (EL) scanning technique. 

## 2. Experimental Section

**Figure 1:**
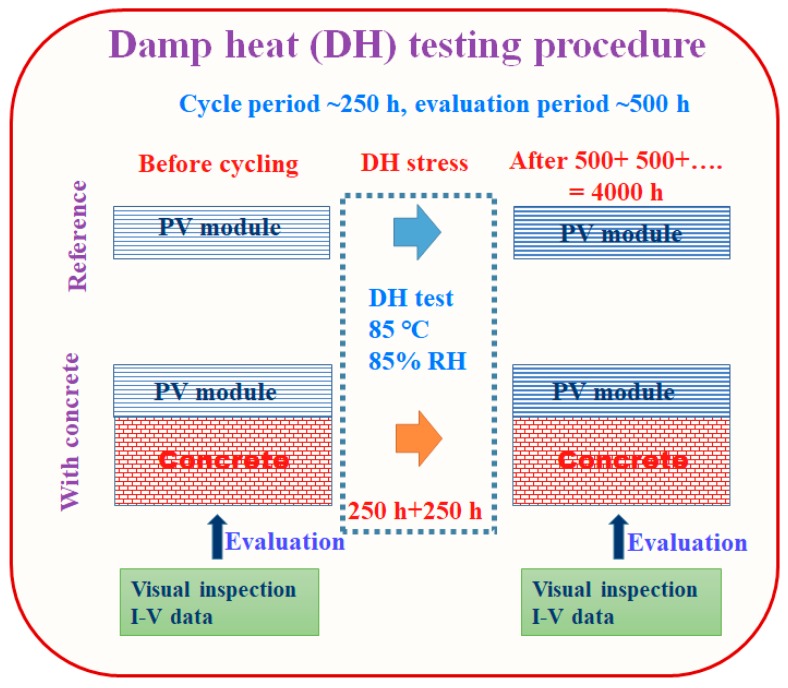
Schematic for the procedure used for Damp-Heat (DH) stress test.

**Figure 2:**
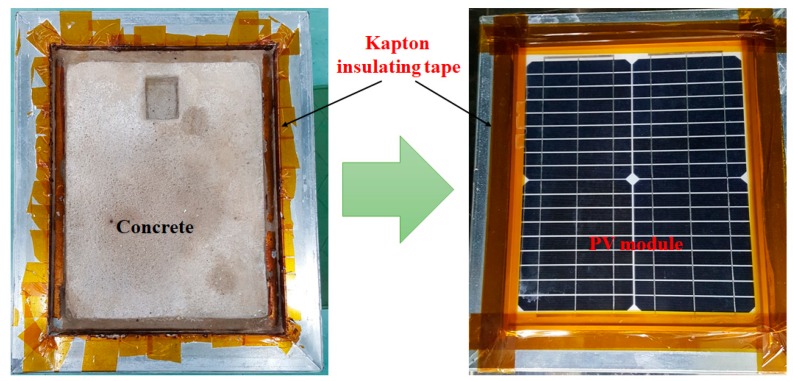
Optical image of (**left**) metallic isolation box with concrete (concrete slab) and (**right**) photovoltaic (PV) module mounted on concrete slab.

Monocrystalline PV modules (~20 W) were used for this study. The rigid modules included 36 cells, with an Al frame (module area = 1540 cm^2^, cell area = 31.2 cm^2^). DH stress was conducted at a temperature of 85 °C and a relative humidity of 85% for a DH stress cycle of 250 h (Figure 1). The DH test was conducted using an environmental chamber (Model: DS-323MHPS-153, M/s. DIMOSTECH, Incheon, Korea). Solar PV modules without concrete (3 modules), where the backside of the module was exposed to the environment, were used as a reference. However, the backside of the PV modules was covered with a concrete-filled metallic isolation box (4 modules, double-wall construction, with an insulating material filling the gap between the walls). After fitting the PV module with the isolation box, the gap between the edges of the module and the box was sealed with Kapton (polyimide) tape. The tape protected the backside of the module from moisture and heat (Figure 2). After each cycle (250 h), the PV modules were visually inspected using optical photographs of the front and rear surfaces. The EL scanning technique was used to obtain EL images using the EL system (Model: TE-2000, M/s. TNETECH, Gyeonggi-do, Korea) by applying a short circuit current.

Illuminated *I–V* characterization was conducted after each 500 h of DH stress. The illuminated *I–V* characteristics were obtained with a Keithley 2420 System SourceMeter using a solar simulator M/s Spire (Model: SPI-SUN SIMULATOR 4600SLP, The Hague, Netherlands) at 25 °C under 100 mW/cm^2^ illumination intensity of a simulated AM 1.5 Global solar spectrum. The intensity of illumination was measured using a reference silicon solar cell obtained from PV Measurements, Washington, DC, USA.

## 3. Results and Discussion

### 3.1. Performance Parameters Analysis

**Figure 3:**
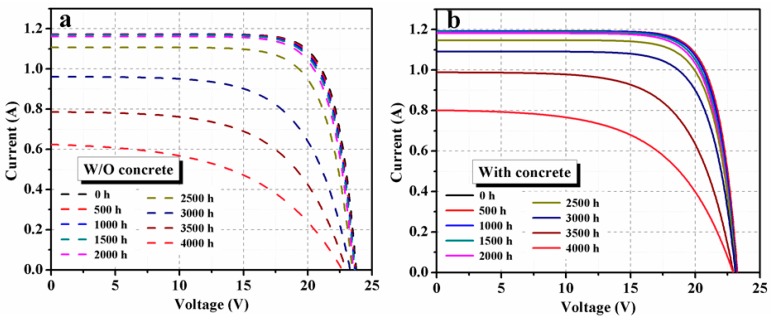
Illuminated *I–V* curves for various DH stress duration of PV module (**a**) without concrete, and (**b**) with concrete.

**Figure 4:**
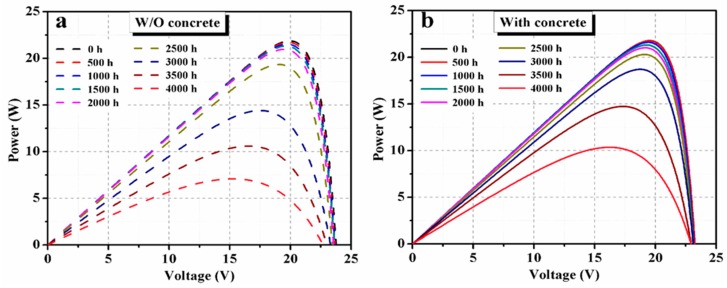
Illuminated *P–V* curves for various DH stress duration of PV module (**a**) without concrete, and (**b**) with concrete.

**Figure 5:**
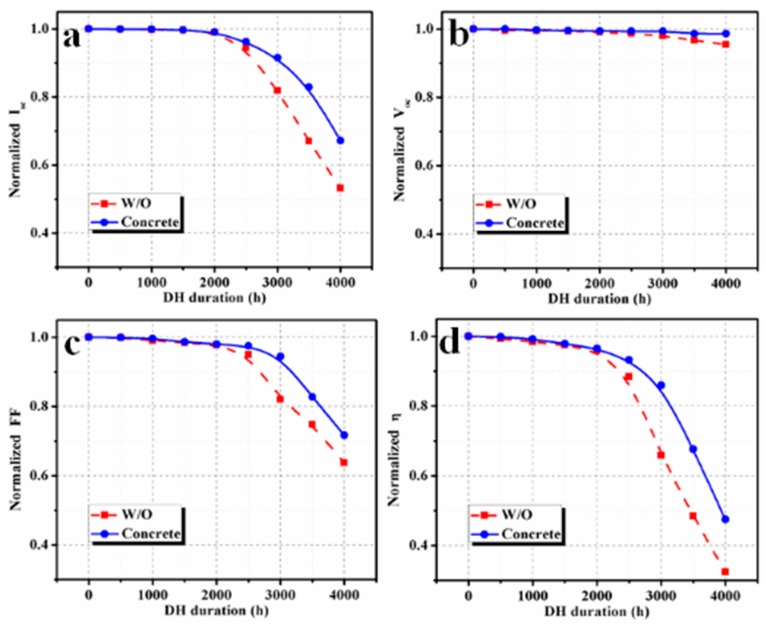
The variation of (**a**) short circuit current, (**b**) open-circuit voltage, (**c**) fill factor and (**d**) conversion efficiency with DH stress duration.

Four PV modules were used with the concrete slab, with three PV modules as references. Similar degradation was observed in each group. Here, one PV module in each group has been analyzed in detail. Moisture normally diffused into solar modules through their breathable back sheets or ethylene-vinyl acetate (EVA) sheets. In solar roadways, the top layer of the PV modules will be high-grade glass, so the backside of the PV module should be protected by moisture absorbent materials. Concretes will be a cheaper option to provide both mechanical strength and protection against the moisture or humidity, and even, to some extent, short flooding with water. Here, the PV modules were fixed on a concrete slab using the isolation box and Kapton insulating tape, and the results were promising. Compared to a reference PV module (without a concrete slab on the back), normalized values of the output power were improved when concrete was used. *I–V* curves of PV modules monitored without (reference PV module) and with concrete for different DH durations are shown in Figure 3a,b, respectively. Corresponding power*–*voltage (*P–V*) curves are shown in Figure 4. Performance parameters that were extracted from the *I–V* data are presented in Figure 5.

There was no significant change in *V_oc_* in either PV module until 1500 h in a DH chamber. However, as the duration increased further, the *V_oc_* value of the reference module started to degrade considerably compared to the *V_oc_* value of the PV module with concrete. After 4000 h, the obtained degradation in *V_oc_* was 4.5% and 1.4%, respectively, for reference and concrete-PV modules. Compared to the concrete-PV module, both *I_sc_* and *FF* were reduced faster up to 2000 h in the reference PV module. The normalized value of *η* of the PV module with concrete was reduced to 96.5%, 85.9% and 47.5% for the DH duration of 2000, 3000 and 4000 h, respectively. On the other hand, the *η* value of the reference PV module was reduced to 95.9%, 65.9% and 32.4% of its initial value for DH duration of 2000, 3000 and 4000 h, respectively.

**Figure 6:**
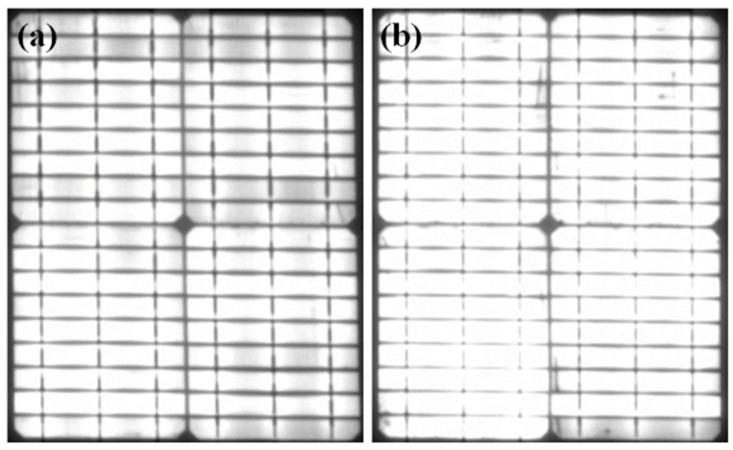
Electroluminescence images of PV modules (**a**) without concrete, and (**b**) with concrete before DH stress test.

**Figure 7:**
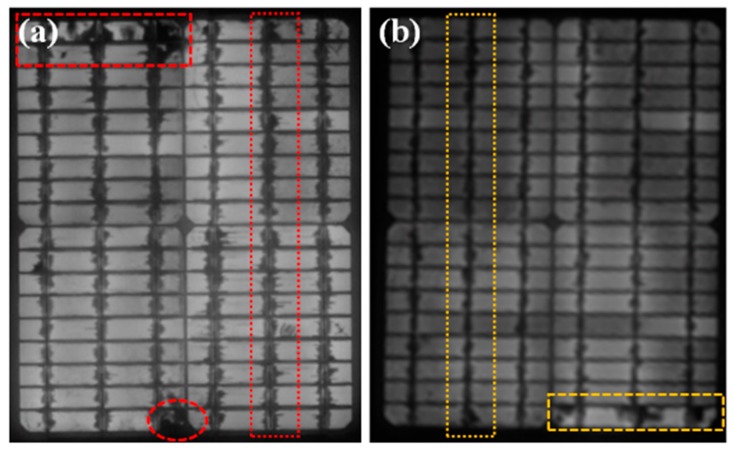
Electroluminescence images of PV modules (**a**) without concrete, and (**b**) with concrete after DH stress duration of 2000 h.

EL results demonstrate that there was no defect in both the PV modules before the DH stress test (Figure 6). The *I_sc_* degradation of both PV modules was very slow for up to 2000 h of DH exposure. However, the degradation of the rate of *I_sc_* of PV module with concrete was slightly lower than the reference PV module in this duration. Figure 7 shows the EL images of the reference PV module and PV modules with concrete after a DH stress of 2000 h. Defective regions are indicated by the red dotted/dashed rectangles/circle (Figure 7a). A lower number of defects was found in the PV modules with concrete (Figure 7b), which revealed that the impact of DH stress on the PV module with concrete was smaller than that of the reference module. With a further increase in DH duration, the *I_sc_* degradation rate for the reference PV module was faster than that of the PV module with concrete. After a DH stress of 3000 h, the *I_sc_* values were reduced to 81.9% and 91.5% of their initial values, respectively, with reference to the PV module and PV module with concrete. The DH stress caused inhomogeneous aging in the PV modules, which reduced the current generation. Hence, *I_sc_* was reduced with DH stress [1]. Finally, after 4000 h in a DH chamber, the *I_sc_* values of the reference and PV module with concrete were reduced to 53.2% and 67.2%, respectively. This shows that for 3000 and 4000 h of DH exposure, the *I_sc_* value was reduced by 9.6% and 24.0%, respectively, when concrete was used.

**Figure 8:**
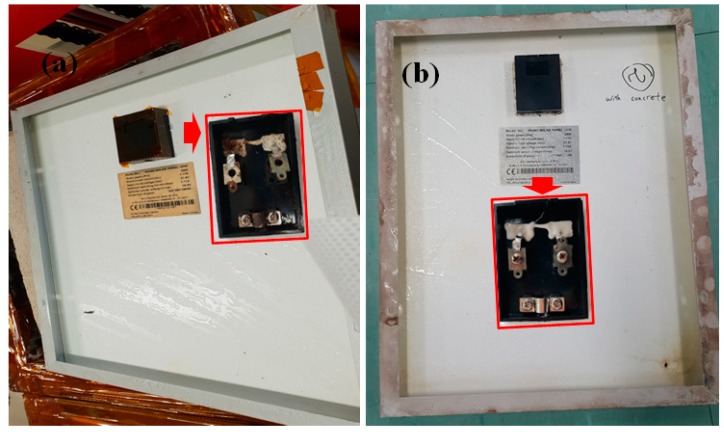
Optical photographs of backside PV modules (**a**) without concrete, and (**b**) with concrete after DH stress duration of 2000 h. (Inset optical photographs of junction box and of corresponding PV modules).

Optical photographs of the backside of the PV modules after DH stress of 2000 h are shown in Figure 8. There is rust on the metal connection in the junction box (inset of Figure 8a) of reference PV modules. However, no rust is seen in the junction box of the PV module with concrete (inset of Figure 8b). These results demonstrate that the concrete contained an isolation box protects the backside of PV modules from exposure to moisture.

**Figure 9:**
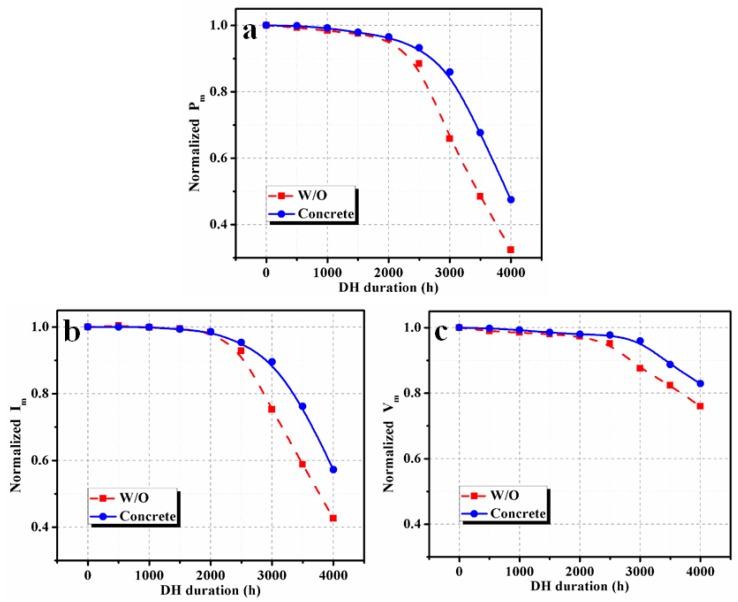
The dependency of (**a**) output power, (**b**) current_,_ and (**c**) voltage at maximum power point on DH stress duration.

Overall, the output power degradation rate in the PV modules with concrete was lower than the reference module. The degradation of maximum power (*P_m_*) is shown in Figure 9a. The corresponding current (*I_m_*) and voltage (*V_m_*) degradation at the maximum power point are shown in Figs. 9b and c, respectively. The degradation rate of *P_m_* in both PV modules was slow until 2000 h of exposure. Normalized *P_m_* values of the PV-concrete module obtained for DH durations of 2000, 2500, 3000, 3500 and 4000 h were 96.5%, 93.2%, 85.9%, 67.6% and 47.5%, respectively. The corresponding normalized *P_m_* values for the reference PV module are 95.9%, 88.4%, 65.9%, 48.5% and 32.4% for DH duration of 2000, 2500, 3000, 3500 and 4000 h, respectively. A similar *P_m_* degradation in the reference PV module over DH duration was observed experimentally and by modeling on previous reports [1,2]. Zhu et al. [1] observed that the degradation rate of a PV module can be reduced using an aluminum moisture barrier with a back EVA sheet.

**Figure 10:**
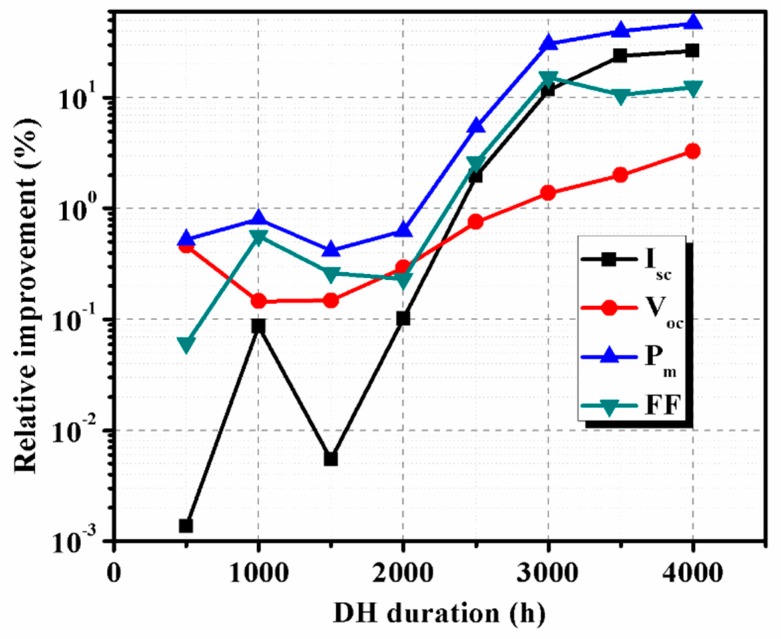
The relative improvement of performance parameters of the PV module with concrete, concerning parameters of PV module without concrete.

It can be seen from Figure 9 that the degradation of *I_m_* had a greater impact on the degradation of *P_m_* than *V_m_* because DH stress reduces the current generation. For a DH duration of 3000 h, the normalized *I_m_* values of reference PV module and PV modules with concrete reduced to 75.2% and 89.6% of their initial values, respectively. Relative enhancement in the performance parameters of the PV module with concrete, concerning the reference PV module, is shown in Figure 10. A significant relative improvement of more than 10% was found for *P_m_* for DH duration beyond 2500 h. This confirms that using concrete reduces the impact of the temperature and humidity on the PV modules, which eventually enhances the performance lifetime of the PV modules.

### 3.2. PV Cell Parameters Analysis

**Figure 11:**
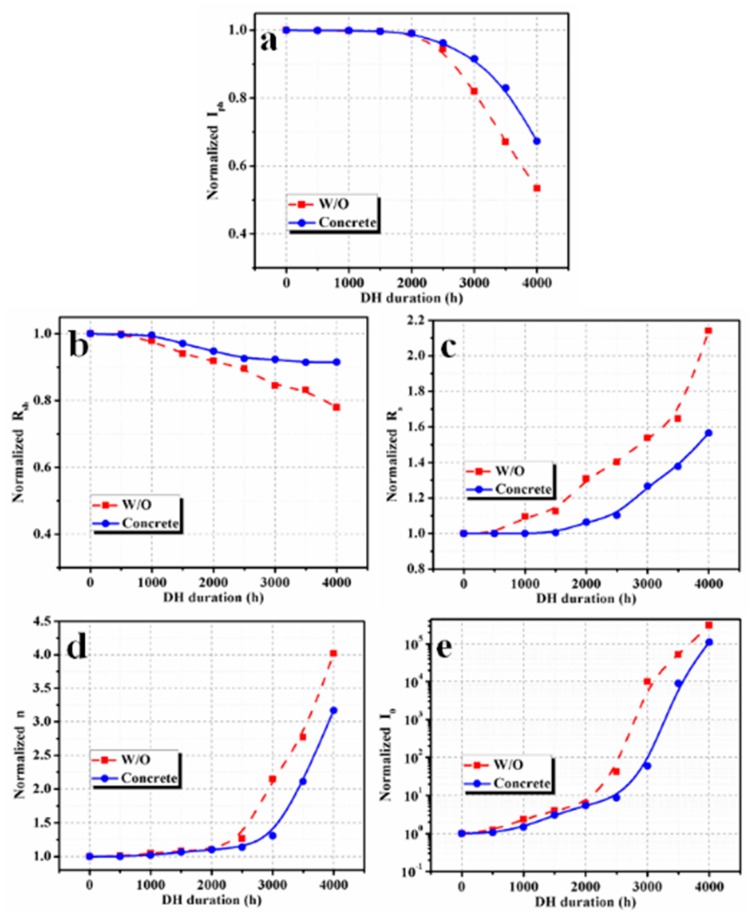
Variation of (**a**) photo-generated current, (**b**) shunt resistance, (**c**) series resistance, (**d**) diode ideality factor, and (**e**) reverse saturation current.

Degradation of the performance parameters can also be explained using the five PV cell parameters. PV cell parameters of the PV modules are compared in Figure 11. Steady-state *I–V* characteristic of p-n junction solar cells, based on a one-diode model [11,12] operating in the first quadrant, is described in [13] as

(1) $$I=\;I_{ph}-I_{0}\left[e^{\left(\frac{V+IR_{s}}{nV_{T}}\right)}-1\right]-\left(\frac{V+IR_{s}}{R_{sh}}\right)$$

The analytical method used in this case [13,14] is based on a single exponential model, which can be used to determine the PV cell parameters at operating temperature *T* under given illumination conditions. This method uses a single *I–V* characteristic curve to determine the five analytical PV cell parameters. The following equations are used to extract the values of *I_ph,_ R_sh_*, *R_s_*, *n*, and *I_0_*, using the values of *I_sc_*, *V_oc_*, *R_sc_*, *R_oc_*, *I_m_*, and *V_m_*.

(2) $$I_{ph}=\;I_{sc}\left(1+\;\frac{R_{s}}{R_{sh}}\right)+I_{0}\left[e^{\left(\frac{I_{sc}R_{s}}{nV_{T}}\right)}-1\right]$$

(3) $$R_{sh}=\;R_{sc}$$

(4) $$R_{s}=\;R_{oc}-\frac{nV_{T}}{I_{0}}e^{-\frac{V_{oc}}{nV_{T}}}\;$$

(5) $$n=\;\frac{\left(V_{m}\mp \;R_{oc}I_{m}-\;V_{oc}\right)}{V_{T}\left\{ln\left(I_{sc}-\;\frac{V_{m}}{R_{sc}}-\;I_{m}\right)-ln\left(I_{sc}-\;\frac{V_{oc}}{R_{sc}}\right)+\left(\frac{I_{m}}{I_{sc}-\;\frac{V_{oc}}{R_{sc}}}\right)\right\}}$$

(6) $$I_{0}=\left(\;I_{sc}-\;\frac{V_{oc}}{R_{sc}}\right)e^{-\frac{V_{oc}}{nV_{T}}}\;$$

Here, *R_sc_* = (dI/d*V*)^−1^ at short circuit conditions (*V* = 0, *I* = *I_sc_*), *R_oc_* = (d*I*/d*V*)^−1^ at open circuit conditions (*V* = *V_oc_*, I = 0), and *V_T_* = k*T*/q (k being the Boltzmann’s constant, and q the elementary electronic charge).

A similar variation in *I_ph_*, as observed in *I_sc_*, is obtained with DH duration, confirming that the DH stress has a decisive effect on current generation. In most cases, the *I_ph_* linearly depends on *I_sc_* [14]. The value of *R_sh_* in the PV module with concrete is nearly constant up to a DH duration of 1000 h and reduces to 92.6% after 2500 h of exposure. With a further increase in DH duration to 4000 h, *R_sh_* is reduced to 91.5%. However, the value of *R_sh_* in the reference module starts a gradual reduction from 500 h and then decreases to 89.6% and 77.9% of the initial value at 2500 h and 4000 h, respectively. Typically, *R_sh_* is a parallel highly*–*conductive path within the PV cell due to local imperfection regions, which contain a large number of traps [15]. This occurs because the traps sink the majority charge*–*carriers or the light*–*generated minority charge*–*carriers [16]. More traps are generated with an increasing DH exposure [17], which results in local inhomogeneity in the traps and enhances the non*–*uniform current flow [18], as well as the leakage current through the p-n junction or from the edge [15]. These traps are more dynamic under low illumination conditions [19]. A low value of *R_sh_* has a detrimental effect on *V_oc_* and *FF*, and thereby module performance, particularly at low intensity [10] or a higher operating temperature [14]. The value of *R_s_* exponentially increases with a rise in DH duration. The variation range of *R_s_* was found to be lower in the PV module with concrete than the reference module, whereas it increased faster in the reference PV module. *R_s_* value of the PV module with concrete increased by 10% after 2500 h, while it increased by 40% for the reference PV module. Moreover, after DH stress of 4000 h, the value of *R_s_* increased by 56% and 114% for PV modules with concrete and reference modules, respectively. The *R_s_* value of a PV module is the combined resistance of the PV cell (base and emitter), front/back contacts, the resistance of solder bonds, and the resistance of junction boxes. The DH stress increased defects in the PV material (Si) and deteriorated contact between Si and metallic contacts. Thus, the overall series resistance was increased. Moreover, the reduction of current generation was also a reason to increase the value of the *R_s_*. *R_s_* severely shrinks the value of *FF* of the cell and thus degrades the cell efficiency. Correspondingly, *n* and *I_0_* values also increased with an increase in DH duration. This severely decreased the *FF* of the PV modules and consequently reduced performance [20]. The value of *n* increased to 3.1 times its initial value for DH duration of 4000 h, while the increment of *n* value was higher for the reference module (4 times for DH duration of 4000 h). *I_0_* values increased to 311,645.4 times and 111,654.9 times for the reference module and PV module with concrete, respectively, for DH duration of 4000 h. *V_oc_* and *FF* values were intensely affected by *n* and *I_0_*, where *I_0_* had a decisive effect on the *V_oc_*. The rise in the value of *n* resulted in a higher *V_oc_* value, while the *FF* was severely reduced with an increase in *n* value [20]. It can be seen that the degradation in the *V_oc_* values are quite low, while the corresponding n values are enormously affected with the DH duration. This is accredited to inhomogeneous ageing of the PV cells and the cell mismatch.

The trend in the variation of the PV cell parameters of the reference PV module was similar to the cell parameters of the PV module without a moisture barrier reported by Zhu et al. [1]. However, in their study, the losses were reduced by the insertion of an aluminum moisture barrier on the EVA back sheet. Again, the metallic isolation box with concrete also worked as an effective moisture barrier and reduced the reduction rate of performance. Concrete slabs absorbed some fraction of the moisture, which diffused through the Kapton tape at the edges of the module and thus effected the power degradation rate.

### 3.3. Measurement Error Analysis

The error in the directly measured quantities are under a level of confidence [21,22]. However, errors propagation method is used to determine the errors in the calculated parameters. The error limits of various parameters are listed in Table 1.

## 4. Conclusions

IEC standard DH stress testing conditions were used to evaluate the PV module’s degradation of the performance parameters on the PV mounted on a concrete slab. PV cell parameters were analyzed to study the loss mechanism. The comparative study showed that using concrete contained in isolation boxes can reduce the rate of performance degradation of the PV module. EL results revealed that fewer defects were created in the PV module with concrete than the reference module without any concrete slab. Normalized *P_m_* values of the PV module with concrete obtained for DH durations of 2000, 2500, 3000, 3500 and 4000 h were 96.5%, 93.2%, 85.9%, 67.6% and 47.5%, respectively. Corresponding normalized *P_m_* values obtained for the reference PV module were 95.9%, 88.4%, 65.9%, 48.5% and 32.4% for DH durations of 2000, 2500, 3000, 3500 and 4000 h, respectively. For the DH duration of 4000 h, the values of *n* increased by 4.0 times and 3.1 times for the reference module and PV module with concrete, respectively. Similarly, the corresponding *I_0_* values of the reference and PV module with concrete increased to 311,645.4 times and 111,654.9 times, respectively. From this preliminary study, it is clear that the concrete back layer plays an important part in the protection of the PV modules from the destructive effects of high temperature and humidity. A more in-depth study is required, and modified PV modules will be introduced for viable solar roadways, which is in progress in our laboratory.

## Figures and Tables

**Table 1:** Error limits of the measured/calculated quantities.

Parameters	Error (%)
*I_sc_* (A), *I_m_* (A), *V_oc_* (V), *V_m_* (V)	0.12
*FF*	0.21
*P_m_*, *η*	0.56
